# Nitrogen content governs thermoelectric performance in TPU/SWCNT composites

**DOI:** 10.1039/d6ra06192c

**Published:** 2026-10-05

**Authors:** Cordelia Zimmerer, Beate Krause

**Affiliations:** a Leibniz-Institut für Polymerforschung Dresden e.V. (IPF), Division Polymer Materials Engineering Hohe Str. 6 01069 Dresden Germany zimmerer@ipfdd.de; b Leibniz-Institut für Polymerforschung Dresden e.V. (IPF), Division Macromolecular Chemistry Hohe Str. 6 01069 Dresden Germany krause-beate@ipfdd.de

## Abstract

The use of the thermoelectric effect is a sustainable form of energy generation, as it allows waste heat to be harnessed to produce electrical energy. This study contributes to the replacement of the expensive and geopolitically risky metals currently used on an industrial scale with inexpensive and more environmentally friendly polymer-based composites. Composites based on singlewalled carbon nanotubes (SWCNTs) are being investigated, in which the polymer – here thermoplastic polyurethane (TPU) – modifies the thermoelectric properties of the SWCNTs. Three different types of TPU were selected by the Shore hardness, and the composition of the soft segments. The quantitative characterization of the TPU composition was performed using infrared spectroscopy (IR). This allowed the urethane, ester, and ether content to be determined. SWCNT contents ranging from 1 to 5 wt% were incorporated into the TPU grades *via* melt-mixing. It can be shown for the first time that an increasing proportion of urethane groups in the TPU leads to a reduction in the Seebeck coefficient from around 40 µV K^−1^ up to 10 µV K^−1^, which indicates an n-type doping effect. Furthermore, it has been shown that the contents of ether and ester groups have a minor effect on thermoelectric properties. A power factor of 0.1 µW m^−1^ K^−2^ could be achieved, which is significantly higher than the power factor previously reported for TPU/MWCNT composites.

## Abstract 2

Comparison of ester/ether/urethane segment ratios in melt-mixed TPU/SWCNT composites, whereby TPU direct interact with SWCNTs; the thermoelectric effect is linked to the hard-to-soft ratio, where nitrogen in urethane lowers the Seebeck coefficient.

## Graphical abstract

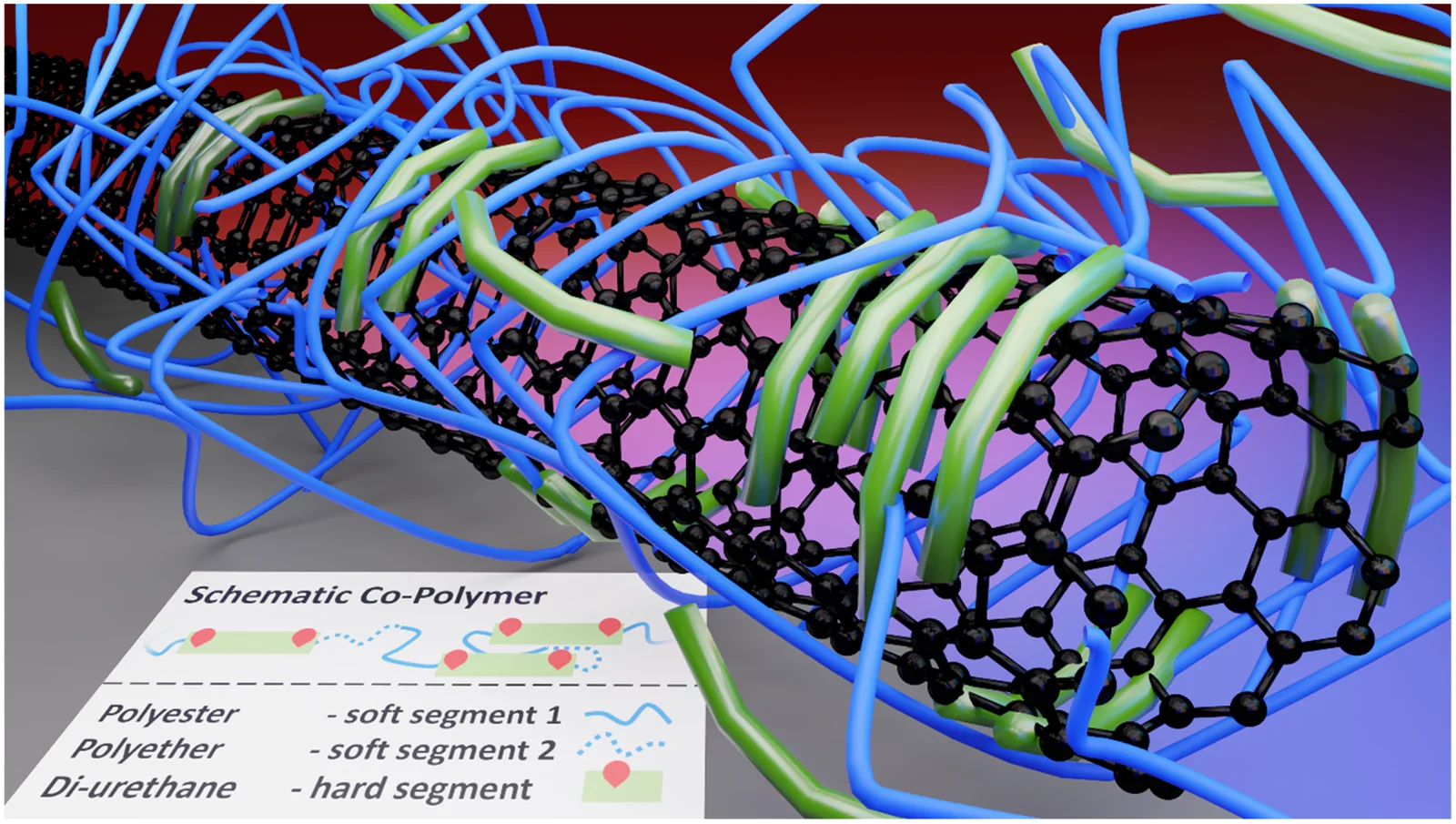

## Introduction

Carbon allotropes are pivotal materials for sustainable energy technologies from nanoscale systems to macroscopic carbon fibers. In polymer composites, they provide lightweight, flexible, and processable platforms for wearable thermoelectrics, soft electronics, smart textiles, self-powered sensors, e-skins, and distributed IoT devices.

Current approaches often rely on chemical functionalization to improve compatibility or dispersion, adding synthetic complexity, cost, and potential damage to the carbon electronic structure. A more powerful yet underused strategy is to design the polymer–carbon interface itself as an active functional interphase. Non-covalent molecular interactions are used to modulate electronic transport without sacrificing nanotube integrity or scalable processing.

To demonstrate this concept, thermoplastic polyurethane/single-walled carbon nanotube (SWCNTs) composites and approaches from interface science, nanocarbon physics, polymer chemistry, and materials engineering are used, because the concept requires an integrated understanding of molecular interactions, charge transport, and composite processing. It establishes polymer chemistry as a direct tool to tune thermoelectric behavior through smart material pairing, enabling the formation of solvent-free, scalable, recyclable, and mechanically compliant carbon–polymer energy materials.

TPU is particularly relevant because its segmented molecular architecture offers distinct chemical motifs, such as urethane, ether, and ester groups, that may interact differently with the nanotube surface. These interactions are expected to occur primarily at the polymer–nanotube interface and may affect the local electronic environment of the SWCNTs without requiring covalent modification.

Investigation and understanding such effects require viewing the composite not only as a mechanically reinforced polymer filled with conductive nanomaterials, but as an interfacial system in which molecular interactions, charge transport, and processing-induced network formation are closely coupled. From this perspective, variations in TPU composition can be used as a tool to probe whether non-covalent polymer–nanotube interactions contribute to changes in thermoelectric behavior.

The thermoelectric effect describes the conversion of heat to electrical energy. To quantify this effect, the Seebeck coefficient *S* is calculated as the quotient of the generated thermoelectric voltage *U* to the temperature difference d*T*. As further value, the power factor PF is calculated as the product of the square of the Seebeck coefficient and the volume conductivity *σ*.^1^ Next to the traditional use of metal-based materials, carbon nanotubes and polymer/carbon nanotubes (CNTs) composites prepared by melt-mixing are also suitable as thermoelectric materials.^2,3^ CNTs (as single-walled CNTs (SWCNTs) or as well as multi-walled CNTs (MWCNTs)) generally have a p-type character of electrical conductivity,^3^ unless nitrogen was introduced into the structure during synthesis.^4,5^ Both, the type of CNT used and the type of polymer have a significant influence on the properties of the composite material. The thermoelectric properties of the composite are always based on the thermoelectric properties of the CNTs. The surrounding polymer merely modifies the properties of the CNTs, as it has no electrical conductivity of its own. However, the polymer can shift the energy level of the CNTs, which is described as a p- or n-doped effect depending on the direction. This has been shown for cellulose^6^ as well as for various thermoplastic polymers.^2^ On the other hand, it was shown that the incorporation of a single CNT type into different polymers leads to different Seebeck coefficients because the polymer structure differently interacts with CNT material on molecular level.^2^

Positive *S*-values were determined for PBT-, PVDF-, PP-, PC-,^2^ PMMA-,^7^ PEEK-,^8^ PCL-, and PLA-based^9^ composites with SWCNT Tuball. However, when the same SWCNTs were incorporated into ABS-, PA6-, PARA- or PA66-based composites, negative Seebeck coefficients were obtained.^2^ This demonstrates that the polymer matrix has a strong influence on the thermoelectric properties of the SWCNT network. Importantly, this shows that the generation of n-type composites is obtainable without the use of further additives.^10^

In the field of SWCNTs modified *via* dip-coating approach,^11^ aerosol-doping,^12^ and solution-mixing processes,^13^ various studies have also been conducted to alter the Seebeck coefficient of CNTs.^14,15^ Nonogucchi *et al.*^16^ reported that next to phosphine derivates several amine-, and imine-containing molecules (*e.g.* poly(ethyleneimine) (PEI), poly(vinylpyrrolidone) (PVP)) are able to convert SWCNT from thermoelectric p- to n-type which is in agreement with the findings of Piao *et al.*^10^ Also Xia *et al.*^17^ described that the CNT treatment with a PEI solution leads to a change of the *S*-value from 76 µV K^−1^ to −72.4 µV K^−1^.

The polymers ABS as well as PA6, PARA and PA66 or PEI have in common that their polymer chain contains nitrogen as nitrile, amide or amine groups. These functional groups possess different reactivity, polarity *etc.*, but have electron-rich regions due to the higher electronegativity value and a lone electron pair of nitrogen (N) in relation to the coupled carbon forming dipolar structures. The spatial arrangement of N-containing functional groups can induce different electronic interactions, *e.g.* share electron density by doping of the CNTs.^18^ However, the SWCNTs were also incorporated into PA12. The PA12 composite with 2 wt% SWCNTs exhibited a positive Seebeck coefficient of 23.6 µV K^−1^ (0.61 S m^−1^, 3.4 × 10^−4^ µW m^−1^ K^−2^). Compared to PA6, PA12 contains only half as many amide groups. It is assumed that the lower amide group content of PA12 is the reason why the Seebeck coefficient is reduced to only slightly positive values compared to the neat SWCNT powder, rather than becoming negative as is the case with PA6.

From the results on ABS and the various PA types, it was concluded that the nitrogen content plays a major role in the doping of SWCNTs and that a certain content of a nitrogen-containing group is necessary for switching from p-type to n-type.

Various studies in the literature demonstrate that polymers can attach themselves to SWCNTs, thereby resulting in the non-covalent functionalisation of the SWCNTs.^19–21^

Molecular dynamics (MD) simulations can be used to predict the conformation of polymer chains on CNT surfaces. Such MD studies often show that flexible polymer chains do not necessarily form a perfect helical structure, but rather adsorbed segments, loops and partially coiled conformations and are focused also on adsorption behaviour and the energetics of polymer–CNT compatibility.^22–24^ The simulations suggest that polymers with less flexible main chains are more likely to wrap around the SWCNTs than those with flexible main chains. Aromatic groups along the main chain appear to be more prone to π–π interactions, whereas aliphatic side chains can hinder these interactions. There is very little literature available on the group of TPU types, examined here, that investigates how the segments of the TPU molecule attach to SWCNTs. This is due to the highly heterogeneous and complex molecular structures of TPUs, which depend heavily on the starting monomers. In the case of TPU, it is expected that the various segments will adsorb onto CNTs in different ways. The polyether soft segment could interact *via* van der Waals interactions and flexible adsorption. van der Waals and dipole interactions *via* ester groups are possible for polyester soft segment. Aromatic diisocyanate are able to interact *via* strong π–π or π-CH interactions. Hydrogen bonding amongst themselves is possible for urethane groups. Buffa *et al.*^25^ described that SWCNT addition to TPU leads to an increase of glass transition temperature of the hard segments and suppressed their crystallization. It can be summarised that it is almost impossible to draw any conclusions about the interaction between the TPU chains and the SWCNTs for the grades investigated in this study, as the effects of the various chain segments vary considerably.

Consequently, the analysis of the chemical composition—along with the conclusions regarding the proportions of ether, ester and urethane present—provides a basis for identifying correlations between the structure of the TPUs and the thermoelectric properties of the TPU/SWCNT composites. In order to investigate this hypothesis, a further N-containing functional group – urethane group – R–O–(C=O)–NH–R, within three different TPU types was analysed in the present study. The chemical compositions of the soft segments were varied between polyester and polyether. In addition, different ratios of soft to hard segments were used, which is reflected in the Shore hardness, a measure of polymer elasticity. The nitrogen content was quantified using infrared spectroscopic (IR) analyses. A correlation between the nitrogen content of the TPU types and the Seebeck coefficient is to be examined.

Therefore, the aim of this study was to characterise, understand, and ultimately predict the influence of the matrix material on the thermoelectric properties of the composite. Particular emphasis was placed on the chemical composition of the matrix and on how specific functional groups may affect the filler component. Commercial thermoplastic polyurethanes were selected as model matrices because they differ in both the type and relative amount of polar functional groups. The Seebeck coefficient was used as the primary thermoelectric response parameter and was evaluated as a function of matrix composition. IR spectroscopy was employed as a rapid, accessible, and cost-effective analytical method to identify and compare the IR-active polar groups within the TPU matrices. This approach is intended to support a broader applicability of the model, including the assessment of commercially available matrices with incompletely disclosed compositions or matrix blends.

## Materials and methods

### Materials

Three different grades of Thermoplastic Polyurethane (TPU) under the trade name Elastollan® from BASF SE (Ludwigshafen, Germany) were used. Grade 1185A10 is a thermoplastic polyether polyurethane elastomer with a density of 1.12 g cm^−3^, a Shore hardness of A87 (or D36) and a melt flow rate at 36.1 g/10 min (190 °C, 21.6 kg). Grade C85A10 is a thermoplastic polyester polyurethane elastomer with a density of 1.19 g cm^−3^, a Shore hardness of A87 (or D36) and a melt flow rate at 36.8 g/10 min (200 °C, 21.6 kg). Grade C74D50 is a thermoplastic polyester polyurethane elastomer with a density of 1.25 g cm^−3^ and a Shore hardness of D73. It was not possible to measure the melt flow rate for C74D50 under comparable conditions (200 °C, 21.6 kg), as the polymer did not melt in the capillary.

The carbon nanotubes selected for this study were SWCNT Tuball™ (OCSIAl, Luxembourg) with a carbon content of 75%.^26,27^ The selection was based on a former study comparing different kinds of CNTs.^2^ RAMAN characterisation of this CNT type are published in ref. 28 and XPS characterisation in ref. 29. Thermoelectric parameters of the SWCNT powder were described in ref. 2. This kind of SWCNTs is abbreviated as Tuball.

### Composite preparation

The composites were prepared using a conical twin-screw micro-compounder Xplore 15 (Xplore, Sittard, The Netherlands) with a volume of 15 cm^3^. The TPUs and SWCNTs were dried at least 6 hours overnight at 100 °C or 80 °C under vacuum, respectively. The SWCNTs were filled into the microcompounder alternately with the polymer granules. The melt mixing was performed at 220 °C (1185A10, C85A10) or 230 °C (C74D50) and 250 rpm for 5 min. The obtained extruded strands were cut into small pieces and compression-moulded into plates (60 mm diameter, 0.5 mm thickness) using the hot press PW40EH (Otto-Paul Weber GmbH, Germany) at the processing temperature for 1.5 min (50 kN max. force, 2.5 min pre-heating).

### Characterization

For characterisation of the macrodispersion of the SWCNT in the TPU matrix, the composite filled with 1 wt% SWCNT was selected for investigation by transmission light microscopy using a light microscope (Olympus BX 53M-RLA) equipped with an Olympus DP74 camera. The extruded strands were cut using a Leica RM2265 instrument in thin slices with a thickness of 5 µm and were fixed with Aquatex on a glass slide.

The SWCNT nanodispersion in the composites was examined using scanning electron microscopy (SEM) on an Ultra plus microscope (Carl Zeiss GmbH, Germany) operated at 3 kV with a field emission cathode on the cryo-fractured surface. Prior to the test, the samples' surfaces were coated with a 3 nm platinum layer.

The thermoelectric (TE) characterization was carried out on rectangular specimens (length 30 mm, width 5 mm) in a measuring device developed and constructed at IPF Dresden.^30^ The measuring temperature was set to 40 °C with four temperature variations up to ±8 K. More details are given in Krause *et al.*^31^

Sampling: each sample was investigated as doublet: freshly cut and measured at two different local positions.

IR spectra have been recorded with a Vertex 70v (Bruker Corporation, Billerica, MA, U.S.A.) equipped with a sampling accessory Platinum ATR, with diamond crystal, and a DLaTGS detector. Spectrometer-associated Software package OPUS 7.5 was used for spectra pretreatment and data evaluation. Measurement parameters were set to: co-added 100 scans per spectrum, spectral resolution of 4 cm^−1^ and an aperture of 4 mm. Sample contact to the ATR crystal was adjusted by a pressure clamp.

Recorded spectra were averaged for each sample. Background spectra have been collected against air under identical conditions.

Spectra were collected under defined instrumental conditions, subjected to standardized preprocessing: linear baseline subtraction extended by 3 iterations of Opus-internal concave rubber band method. Vector normalization was performed in the spectral range of 4000 cm^−1^ to 900 cm^−1^. Spectra analysis, using fast and simple approaches to extract qualitative and semi quantitative information, was carried out by integration of unsuperimposed bands or deconvolution of band patterns and a subsequent integration of the sub bands resulted in relative quantifications and assignments of functional groups and its relative content.

Spectral range of 3015 cm^−1^ to 2530 cm^−1^ was considered for the structure elucidation and comparison of polymeric backbone and put into relation to the functional urethane groups.

The integration of sub bands for the quantification of the C=O band pattern as in ester groups and as in urethane groups, in parallel for N–H stretching band evaluation for urethane groups, as well as for C–O band assigned to ether groups was performed with the Opus-provided 2-base-points-method.

The normalized and baseline-corrected spectra were evaluated to compare the relative ester and urethane contributions within the spectral regions listed in Table 2. Prior to this analysis, second-derivative spectra were calculated to identify local minima, which were then used to determine the band positions for spectral deconvolution in the C=O stretching region.

## Results

For commercial purposes in general, TPU is formed by the reaction of three components: polyols(long-chain diols), diisocyanates, and short-chain diols. The used polyols molecular structure has a significant influence on certain properties of the TPU. Often polyester or polyether soft segments are used. Through polyaddition, the polyols and short-chain diols react with the diisocyanates to form linear polyurethane. The type of raw educt materials, the reaction conditions, and the proportions of the starting educts determine the TPU properties.

Shore hardness is a measure of a polymer's elasticity and is strongly depending on the ratio of soft and hard segments (Fig. 1). The Shore A and Shore D hardness scales differ primarily in their applications. Shore A is used when softer, more flexible materials are required such as rubber- and silicone-like, while Shore D is designed for harder, stiffer material in applications such as plastics and hard elastomers. In both scales, hardness increases as the number following the letter increases and as the proportion of hard segments increases. However, Shore hardness as sum parameter depends not only on the proportion of hard segments, but also on the type of diisocyanate, the chain extender, the polyol(polyester, polyether) and its molecular weight, the crystallinity of the hard segments, phase separation between hard and soft segments, hydrogen bonding and other additives, fillers and plasticisers. For two of the TPU types examined (1185A10, C85A10), the manufacturer specified a Shore hardness of A87 and D36, respectively. A hardness rating of A87 indicates a flexible yet sturdy and supportive material that is used, for instance, in cell phone cases. In contrast, a Shore hardness of D73 was given for the grade C74D50, indicating a very hard, almost rigid plastic that offers the best resistance to deformation and pressure. Such materials contain a high proportion of hard segments.

**Fig. 1:**
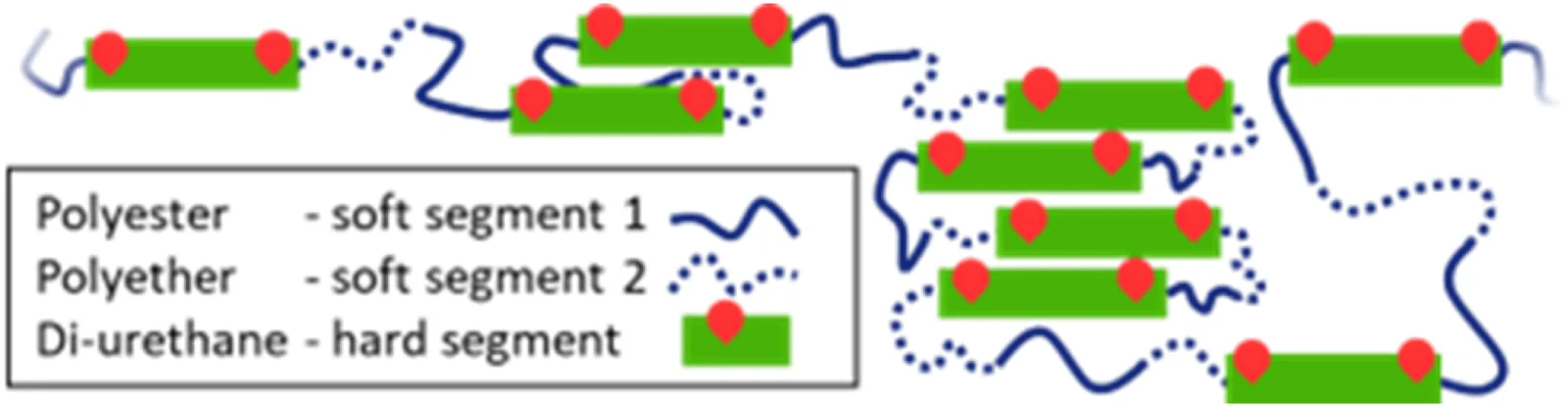
Generalized molecular structures of hard and soft segments of TPUs, red marks symbolize nitrogen-containing urethane groups.

The manufacturer provides very little information on the composition in its data sheets. For example, the grades series distinguishes only between polyester and polyether groups. The proportion of hard segments can only be inferred from the Shore hardness. The only way to undertake the investigation of nitrogen's influence on thermoelectric properties of embedded SWCNTs in TPU is to determine the nitrogen content of polymers is by our own analysis.

### IR characterisation

The characteristic functional units of the TPUs are IR-active and can be identified by their specific band positions. Therefore, the relative evaluation of urethane, and ester or ether contributions of the different polymeric matrices have been internally referenced, because the dielectric and indirect the mechanical properties affect the IR quantification and are listed in Table 1.

**Table 1:** Supplier information and resulting attributes that affect the IR sample measurements

Type	C74D50	C85A10	1185A10
Chemical property: relative functionalization by diisocyanate concluded from the Shore hardness	High	Low	Low
Chemical property: polyole base	Polyester	Polyester	Polyether
Physical properties: relative hardness and density	Hard material, highest density	Soft material, lower density	Soft material, lower density
IR relevant properties for relative spectra recording	Low penetration depth, low materials compression by pressure clamp, varying optical contact	High penetration depth, high materials compression by pressure clamp, less varying optical contact	High penetration depth, high materials compression by pressure clamp, less varying optical contact

Owing to their comparatively high IR absorption coefficients, urethane-, ester-, and ether-related bands can be detected reliably even at relatively low contents. Fig. 2 shows stacked overview spectra of the TPU matrices C74D50, C85A10, and 1185A10, with the most relevant spectral regions and numbered key bands highlighted.

**Fig. 2:**
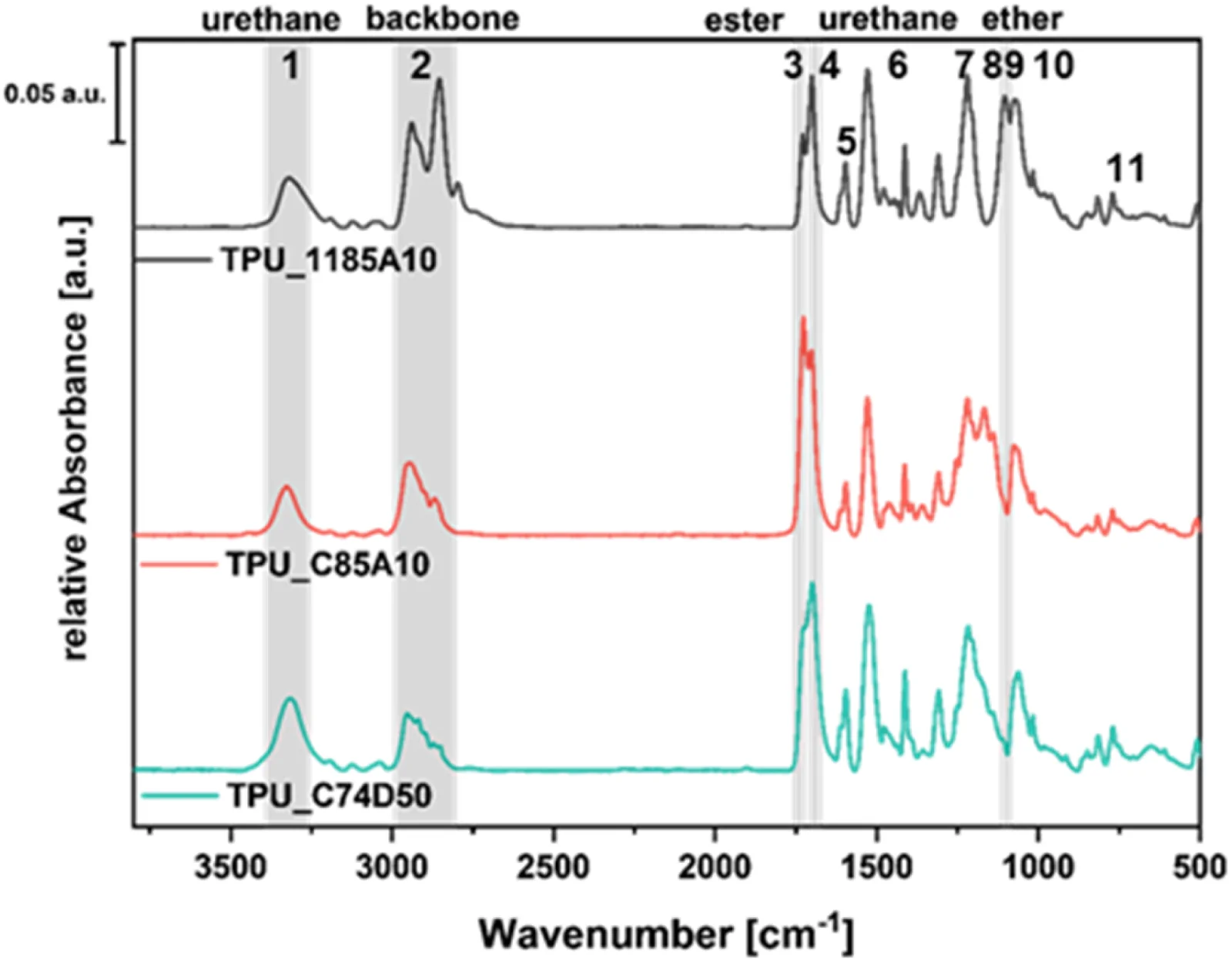
Stacked IR absorption spectra of the TPU matrices C74D50, C85A10, and 1185A10. Relevant spectral regions and characteristic key bands assigned to urethane, ester, and ether functionalities are indicated.

The purpose of the spectroscopic analysis was not to determine absolute concentrations of the individual TPU functionalities, but rather to compare their relative contributions within each matrix. A direct comparison between the samples is complicated by differences in optical and mechanical material properties, including refractive index, extinction coefficients, density, and compressibility. These later parameters can affect the effective probed volume and thus the apparent spectral contribution of individual functional groups.

Therefore, an internally referenced evaluation procedure was applied as an appropriate approximation for relative comparison. The IR spectra of the TPUs in the wavenumber range from 4000 to 400 cm^−1^ are shown in Fig. 2. The highlighted spectral regions contain characteristic features of urethane, ester, and ether groups. The corresponding band assignments are summarized in Table 2.

**Table 2:** Overview of characteristic IR bands and their assignment to functional groups in the TPU matrices

No.	Band position [cm^−1^]	Relative intensity^a^	Assignment, type of vibration	Functional group	Reference	Comments
1	3320	m	*ν*N–H	–O–(C=O)–NH–	32 and 33	Urethane, A^a^ A
2	2960–2736	s–vs	*ν*C–H	–CH_3_; –CH_2_–; –CH <svg xmlns="http://www.w3.org/2000/svg" version="1.0" width="10.400000pt" height="16.000000pt" viewBox="0 0 10.400000 16.000000" preserveAspectRatio="xMidYMid meet"><metadata> Created by potrace 1.16, written by Peter Selinger 2001-2019 </metadata><g transform="translate(1.000000,15.000000) scale(0.011667,-0.011667)" fill="currentColor" stroke="none"><path d="M480 1160 l0 -40 -40 0 -40 0 0 -40 0 -40 -40 0 -40 0 0 -40 0 -40 -40 0 -40 0 0 -40 0 -40 -40 0 -40 0 0 -40 0 -40 -40 0 -40 0 0 -80 0 -80 40 0 40 0 0 40 0 40 40 0 40 0 0 40 0 40 40 0 40 0 0 40 0 40 40 0 40 0 0 40 0 40 40 0 40 0 0 40 0 40 40 0 40 0 0 40 0 40 40 0 40 0 0 40 0 40 -80 0 -80 0 0 -40z M80 480 l0 -80 40 0 40 0 0 -40 0 -40 40 0 40 0 0 -40 0 -40 40 0 40 0 0 -40 0 -40 40 0 40 0 0 -40 0 -40 40 0 40 0 0 -40 0 -40 80 0 80 0 0 40 0 40 -40 0 -40 0 0 40 0 40 -40 0 -40 0 0 40 0 40 -40 0 -40 0 0 40 0 40 -40 0 -40 0 0 40 0 40 -40 0 -40 0 0 40 0 40 -40 0 -40 0 0 40 0 40 -40 0 -40 0 0 -80z"/></g></svg>	34	Methyl, methylene, methine, aliphatic backbone units
3	1732	s	*ν*C=O	–O–(C=O)–	32 and 33	Ester
4	1701	vs	*ν*C=O	–O–(C=O)–NH–	32 and 33	Urethane, A I
5	1600	m	*δ*N–H	–O–(C=O)–NH–	34	
6	1530	s	*ν*C–N, *δ*N–H	–O–(C=O)–NH–	34	Urethane, A II
7	1220	s	*ν*C–N, *δ*N–H	–O–(C=O)–NH–	34	Urethane, A III
8	1200–1120	s	*ν*(C=O)–O–C		32–34	Ester
9	1105	s	*ν*C–O–C		34	Ether
10	1087–1050	m–s	*ν*C–O–(C=O)	–O–(C=O)–NH–	32 and 33	Urethane
11	770	w	Complex	–O–(C=O)–NH–	32	Urethane, A IV

a Amide bands (A): A A, A I, A II, A III, A IV

As a first relative spectral descriptor of urethane-associated contributions, the N–H stretching region was evaluated in the wavenumber range of 3700–3000 cm^−1^. The integrated band area of the N–H stretching vibration revealed broad absorption features and distinct differences in band shape among the investigated polymer matrices.

These differences in N–H band intensity and shape indicate variations not only in the relative urethane-associated spectral contribution, but also potentially in the hydrogen-bonding environment of the hard segments. Therefore, the N–H stretching region was used as an initial descriptor, while further differentiation of urethane and ester contributions was obtained from the carbonyl stretching region.

The relative abundance of urethane groups with respect to the polymer backbone (s. Fig. 3) was first evaluated from the spectral region between 3700 and 2650 cm^−1^. This region contains the N–H stretching vibration of urethane groups as well as the symmetric and antisymmetric C–H stretching vibrations of aliphatic hydrocarbon segments and aromatic components. The calculated band-area integrals are summarized in Table 4. Based on this evaluation, TPU C74D50 exhibits the highest urethane-related spectral contribution, whereas C85A10 and 1185A10 show lower and approximately comparable urethane contributions. The parameter N1 represents the integrated area of the N–H stretching band linearly normalized to a maximum value of 1 and thus allows a first approximate comparison of nitrogen-containing urethane-related groups among the different TPU matrices.

**Fig. 3:**
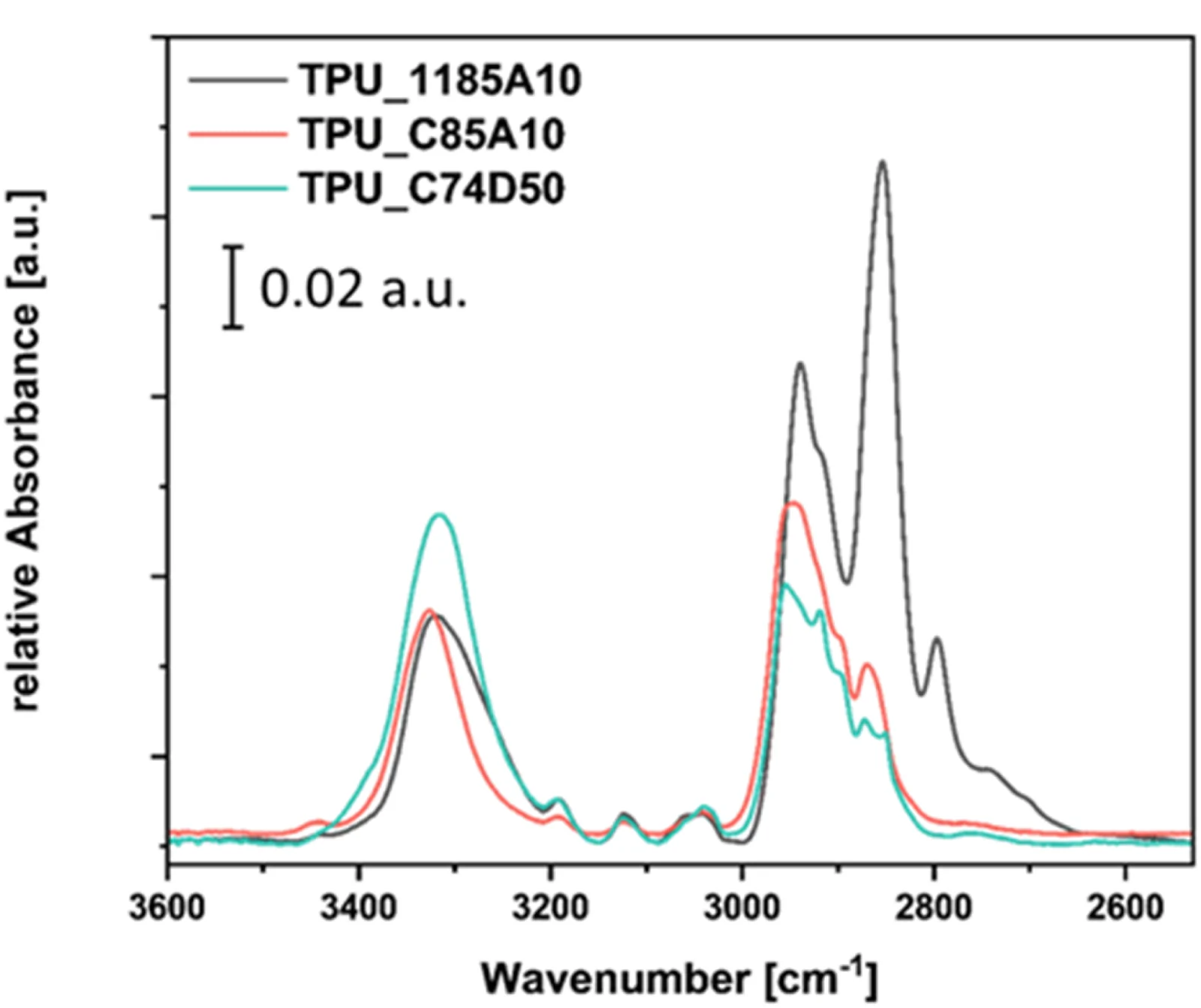
Spectral sections of the IR spectra of C74D50, C85A10, and 1185A10 in the stretching-vibration region after vector normalization of the corresponding full spectra.

However, the N–H stretching region may be affected by sample-specific spectral contributions from O–H groups or amine-containing species that are not part of urethane functionalities. Therefore, the relative urethane contribution was additionally evaluated from the carbonyl stretching region, together with the ester contribution. This second approach provides a more specific assessment of urethane and ester functionalities, although it requires spectral deconvolution of the overlapping carbonyl bands.

The carbonyl stretching vibration appears as a double band with a complex band shape. Deconvolution of this absorption region reveals two dominant components. The band at approximately 1730–1740 cm^−1^ is assigned to the C=O stretching vibration of ester groups, whereas the main band at approximately 1700 cm^−1^ is assigned to the C=O stretching vibration of urethane groups. Additional overlapping bands are present and can be attributed to different associated or secondary bonding states, for example due to hydrogen-bond formation.

To resolve the overlapping carbonyl contributions, the second derivative of the spectra was calculated in the carbonyl stretching region between 1800 and 1650 cm^−1^. In second-derivative analysis, the number and position of local minima provide an estimate of the number and position of overlapping absorption bands in complex band envelopes. For all three TPU matrices, the second derivative shows one dominant sub-band minimum at approximately 1730 cm^−1^ and a second one at approximately 1700 cm^−1^. An additional minor minimum occurs at approximately 1714 cm^−1^ in all samples, while a further weak contribution around 1740 cm^−1^ is observed in two of the three matrices. The resulting band positions and assignments are summarized in Table 3.

**Table 3:** Second-derivative analysis of the complex carbonyl stretching region and assignment of the resolved sub-bands according to Socrates^32^

TPU	Minimum position [cm^−1^] C74D50/C85A10/1185A10	Relative intensity^a^	Functional group	Comments
	1740/1739/—	w	Ester	Free C=O
	1731/1727/1730	s	Ester	Secondary interactions, H-bonds C=O
	1715/1713/1714	w–m	Urethane	Free C–O–(C=O)–NH
	1697/1700/1700	s	Urethane	Secondary interactions, H-bonds C=O

a No fixed unit, qualitative: s = strong, m = medium, w = weak.

According to the commonly observed red shift of stretching vibrations upon hydrogen-bond formation, the intense carbonyl bands indicate that a substantial fraction of the ester and urethane carbonyl groups is present in associated states. Weak secondary minima at higher wavenumbers suggest that a smaller fraction of carbonyl groups may be present in less strongly associated or free states. Consequently, the integration ranges for quantifying the corresponding functional groups must be chosen sufficiently broad to include both associated and non-associated contributions. For the subsequent spectral deconvolution, the sub-band positions identified from the second-derivative spectra were used as initial fitting constraints.

For the quantitative determination of urethane-related contributions, both approaches were compared: evaluation of the N–H stretching region and evaluation of the deconvoluted carbonyl stretching region. The carbonyl-based approach was selected for further analysis because, despite requiring an additional deconvolution step, it provides a higher information content by enabling simultaneous differentiation between urethane and ester contributions (see Table S2) (Fig. 4).

**Fig. 4:**
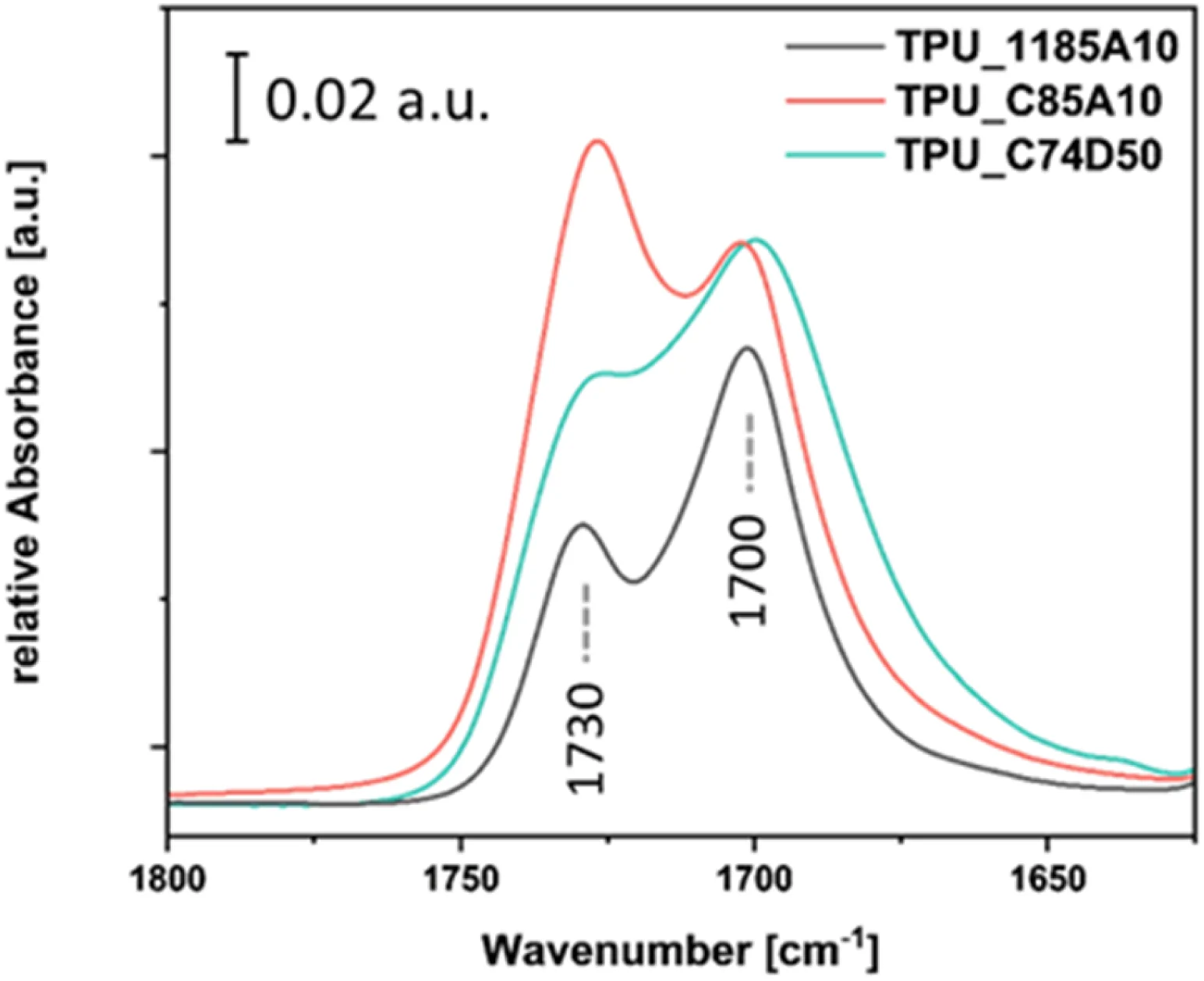
Selected spectral range of the complex carbonyl stretching region used for deconvolution of ester-related C=O bands around 1730 cm^−1^ and urethane-related C=O bands around 1700 cm^−1^.

The quantitative results are listed in Table 4. The normalized parameter N2 describes the urethane contribution obtained from the carbonyl stretching region and linearly scaled to the maximum value of 1. Overall, both spectral approaches yield comparable trends in the relative urethane contributions of the matrices. C74D50 shows the highest urethane-related contribution, whereas C85A10 and 1185A10 exhibit lower values. However, the N2 values of C85A10 and 1185A10 relative to C74D50 are slightly lower than the corresponding N1 values derived from the N–H stretching region. This discrepancy may indicate additional contributions to the N–H/O–H stretching region, for example from O–H-containing species, and supports the use of the carbonyl-based evaluation for a more specific comparison.

**Table 4:** Quantification of the relative urethane, ester, and ether contributions in the investigated TPU matrices (parameter N1 – describes the linearly normalized values of integrals of bands assigned to N–H stretching vibrations; parameter N2 denotes the urethane-related carbonyl contribution linearly normalized to the maximum value of 1. The parameter *R* (x) represents the relative contribution of each functional group to the sum of all evaluated functional-group band areas)

TPU	C74D50	C85A10	1185A10
Integral *ν*N–H	3.2	1.9	2.0
N1	1	0.6	0.6
Integral 1 *ν*C=O (ester)	0.7	2.6	0.5
Integral 2*ν*C=O (urethane)	4.4	2.1	1.8
N2	1	0.5	0.4
Integral 3 *ν*C–O–C (ether)	—	—	1.9
Sum integral 1–3 (functional groups)	5.1	4.7	4.2
*R* (ester)	0.14	0.55	0.12
*R* (urethane)	0.86	0.45	0.43
*R* (ether)	—	—	0.45

The ether contribution was evaluated from the C–O–C stretching region. Fig. 5 shows the corresponding spectral sections of the TPU materials. In this region, the band at approximately 1104 cm^−1^ can be assigned to ether functionality and was used for direct quantification. Since this spectral region also contains broad overlapping contributions from ester and urethane-related vibrations between approximately 1050 and 1090 cm^−1^, the integration limits were selected carefully to minimize contributions from adjacent bands. The ether content was quantified by integrating the band area at 1104 cm^−1^. The corresponding integrals and integration parameters are summarized in the SI: Table S1.

**Fig. 5:**
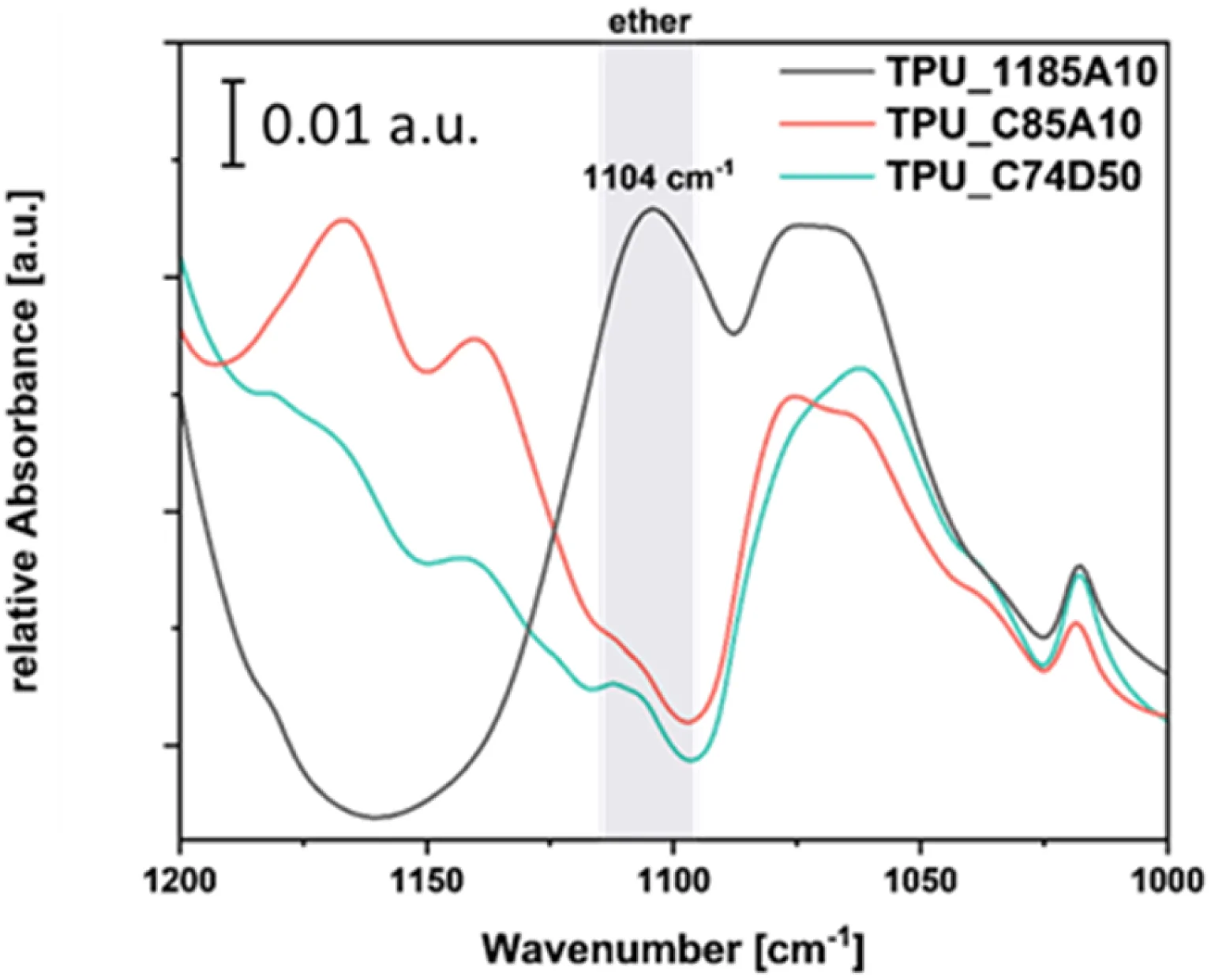
Selected spectral range of the C–O–C stretching region used for the evaluation of ether-related contributions at approximately 1104 cm^−1^. Broad overlapping absorptions of ester and urethane-related groups are observed between approximately 1050 and 1090 cm^−1^.

### Morphological characterisation

The molecular architecture of the polymer TPU matrix, and in particular its relative composition in terms of functional groups such as urethane, ester, and ether moieties, can influence polar and inductive interactions with the filler material. These interactions may modify the physicochemical properties of the filler and, consequently, affect the macroscopic properties of the composite material.

The morphological examination of the composites was performed using transmission light microscopy to assess how well the SWCNTs are distributed within the polymer matrix. Agglomerates as small as the micrometer range can be detected using this method. Fig. 6 shows a compilation of representative images. Individual larger residual agglomerates can be seen in each composite, although there are no significant differences in quantity or number. In all three TPU types, the CNTs are thus distributed in a similarly inhomogeneous manner. The different viscosities of the TPUs, which were quantified on the basis of the melt flow rate, therefore play a minor role in the macroscopic distribution of the SWCNTs. No conclusions can be drawn regarding the nanoscale distribution of the SWCNTs. It can be assumed that sufficient SWCNT bundles or isolated SWCNTs are embedded in the polymer matrix to form an electrically conductive network.

**Fig. 6:**
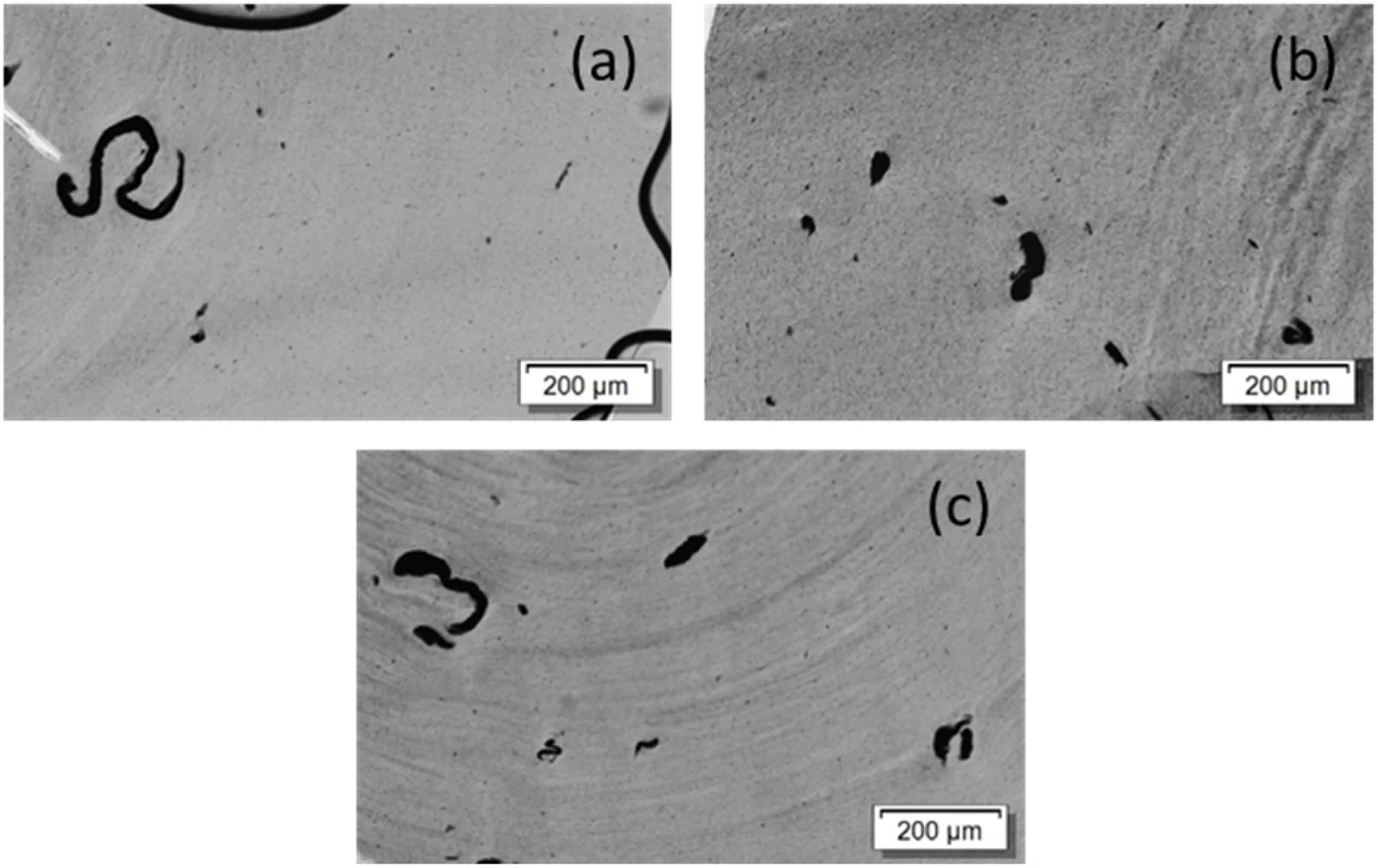
Transmission light microscopy images of TPU composites containing 1 wt% SWCNT Tuball each in (a) 1185A10, (b) C85A10, (c) C74D50.

The distribution of CNTs at the nanoscale was investigated using scanning electron microscopy. Fig. 7 shows the 1 wt% composites in each case. It is noticeable that, for the C85A10 matrix, the SWCNTs are very well wetted and embedded in the matrix, making them barely discernible. For 1185A10, both well-embedded SWCNTs beneath the polymer surface and individual SWCNTs protruding from the matrix can be seen. A large number of isolated SWCNTs can be seen protruding from the surface of the C74D50 polymer matrix. This means that the SWCNTs in this matrix are the least well wetted compared with the other two TPU types. For all three TPU types, a homogeneous distribution of SWCNTs is evident at the nanoscale.

**Fig. 7:**
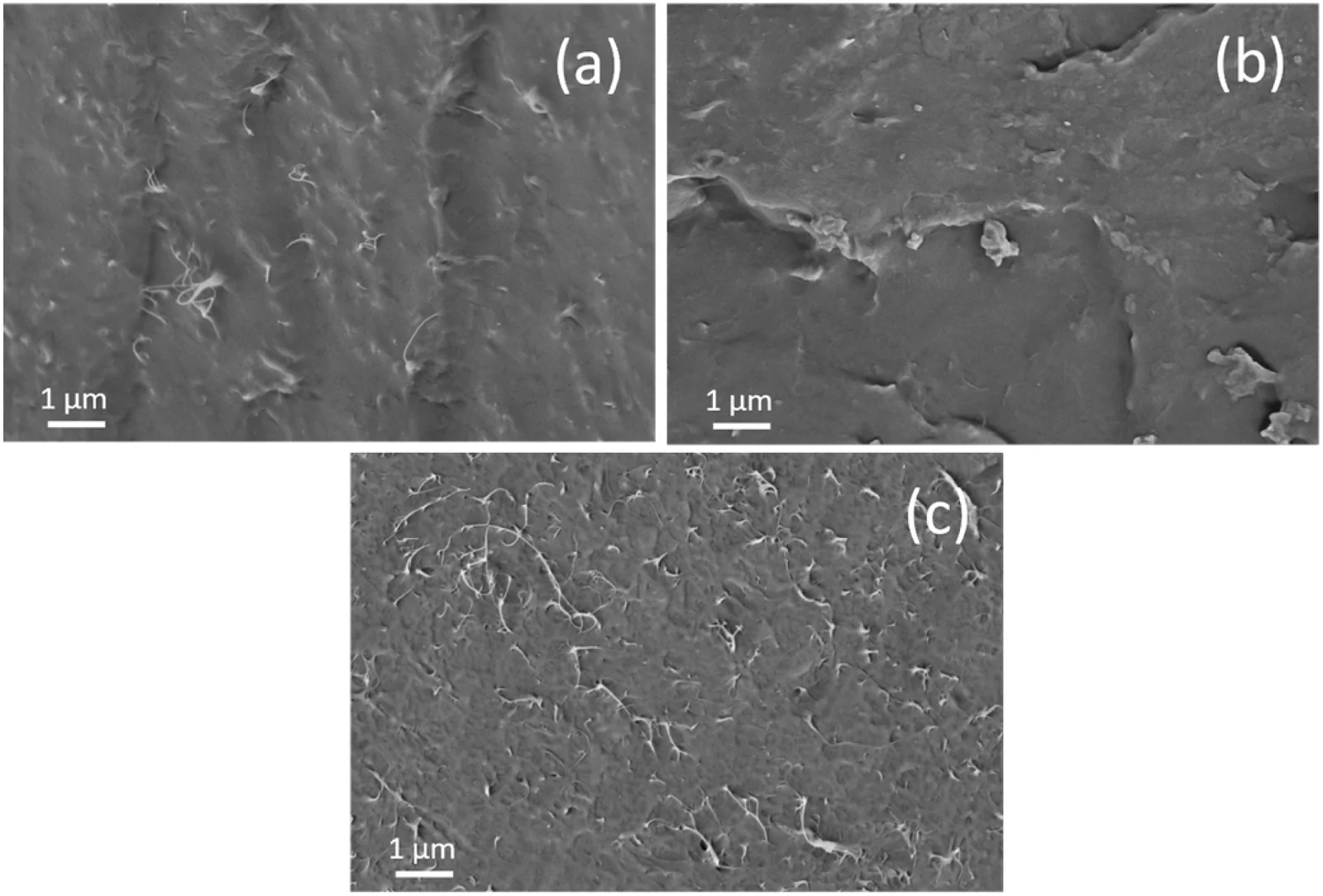
Scanning electron microscopy images of TPU composites containing 1 wt% SWCNT Tuball each in (a) 1185A10, (b) C85A10, (c) C74D50.

SWCNT distribution does not allow conclusions to be drawn about the thermoelectric properties of the entire composite, as the non-covalent interaction between SWCNTs and polymer chains is sufficient for the doping effect. The elongated and curved shape is typical of the Tuball used.^31^

### Thermoelectric properties

For the thermoelectric investigations, SWCNT contents ranging from 1 to 5 wt% were incorporated into the TPU types. These SWCNT contents were chosen because they exceed the electrical percolation threshold. In ref. 35, it was reported that TPU grade 1185A10 exhibits electrical conductivity starting at 1 wt% MWCNT NC7000 from Nanocyl S.A. (Belgium).

As expected from other authors,^35–37^ for all three kinds of composites the volume conductivity raised with SWCNT content (Fig. 8a). The highest values were determined for the C74D50 grade with the highest melt flow rate. The C74D50 matrix exhibited the highest melt viscosity compared with the other two TPU grades. It can therefore be assumed that, during melt mixing, the highest shear forces were applied on the SWCNTs in the C74D50 matrix, resulting in a more pronounced individualisation of the SWCNT bundles. For SWCNT Tuball in poly(methyl methacrylate), it has already been shown that higher shear forces lead to higher electrical conductivity.^7^

**Fig. 8:**
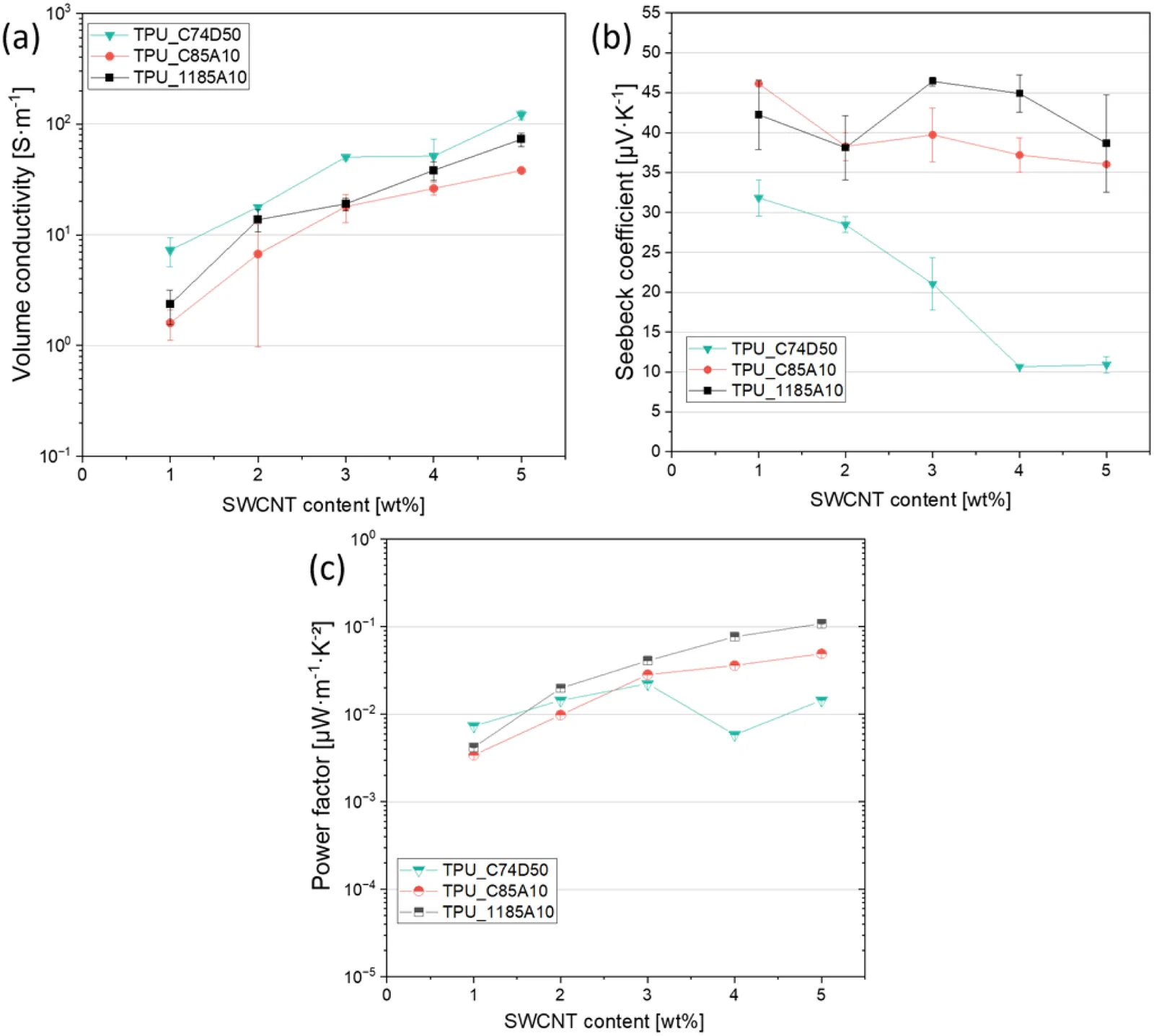
Results of thermoelectric characterization of TPU/SWCNT composites: (a) volume conductivity, (b) Seebeck coefficient *S* and (c) power factor PF depending on SWCNT weight fraction in polymer matrices of different TPUs.

In Fig. 8b, the Seebeck coefficients are shown for all composites. It is remarkable that values around 40 µV K^−1^ were determined for the composites based on C85A10 and 1185A10. This is in the range of the Seebeck coefficient of the Tuball powder, which was determined to be 39.6 µV K^−1^.^2^ This indicates that these two TPU grades do not have a significant effect on the thermoelectric properties of SWCNTs. In contrast, significantly lower Seebeck coefficients are measured for the C74D50 composites. Even with just 1 wt% SWCNT, the *S*-value is 31.8 ± 2.2 µV K^−1^. As the SWCNT content increases, the Seebeck coefficient continues to decrease to 10.7 ± 0.1 µV K^−1^ (4 wt% SWCNT) and 10.9 ± 1.0 µV K^−1^ (5 wt% SWCNT). This trend in values clearly indicates a n-type doping effect of the TPU C74D50 on the SWCNTs, as the Seebeck coefficient is reduced but is still a p-type material.

The calculated PFs increase with the Tuball content (Fig. 8c). The highest power factor was determined for the composites based on 1185A10 and ranges from 0.004 µW m^−1^ K^−2^ to 0.109 µW m^−1^ K^−2^. Slightly lower PF values are calculated for the C85A10 composites, ranging from 0.003 µW m^−1^ K^−2^ to 0.049 µW m^−1^ K^−2^. The lowest PF values are calculated for the C74D50 composites with 4 wt% SWCNT content. This reflects the low Seebeck coefficient at 4–5 wt% SWCNT, even though the volume conductivity reached the highest values when comparing the three TPU types. The PF values of C74D50 composites varied between 0.006 µW m^−1^ K^−2^ at 4 wt% SWCNT and 0.022 µW m^−1^ K^−2^ at 3 wt% SWCNT.

It is difficult to compare these values with those reported in the literature, as there is only one other publication by Tzounis *et al.*^38^ on melt-mixed TPU/CNT composites. In that study, MWCNTs from Nanocyl (NC7000 and long MWCNTs) were incorporated into TPU type 1185A. For composites containing 1–5 wt% MWCNT, Seebeck coefficients of 8–9 µV K^−1^ (NC7000) and 18–19 µV K^−1^ (long MWCNT) were determined. The Seebeck coefficient of NC7000 powder is reported as 6.3 µV K^−1 2^. This means that, here too, the *S*-values of the composite and the MWCNT powder are very similar. No value is reported for the long MWCNT. Unfortunately, the C74D50 type was not used in this study, so that a comparison can be made between different types of TPU. Here, the PF values range from 10^−7^ µW m^−1^ K^−2^ (1 wt% NC7000) to 10^−3^ µW m^−1^ K^−2^ (2.5–5 wt% NC7000) and are thus lower than the values reported in this study. Table 5 provides an overview of the volume conductivities, Seebeck coefficients *S* and power factors PF that can be achieved in thermoplastic composites using the SWCNT Tuball used here. For the purpose of comparability, composites containing approximately 2 wt% were listed. In this study, PF values between 0.01 µW m^−1^ K^−2^ and 0.02 µW m^−1^ K^−2^ were determined for 2 wt% Tuball. This is within a similar range to that of the majority of composites (Table 5). However, with the polymers PMMA, PCL and PLA, PF values are achieved that are one decade higher. For PLA/3 wt% Tuball a power factor of 0.57 µW m^−1^ K^−2^ was reached.^9^

**Table 5:** Comparison of thermoelectric properties of thermoplastic composites filled with around 2 wt% SWCNT Tuball

Polymer	Vol. cond. [S m^−1^]	*S* [µV K^−1^]	PF [µW m^−1^ K^−2^]
PP + 2 wt% (ref. 39)	8 × 10^−4^	35.6	0.010
PBT + 2 wt% (ref. 2)	18.6	58.2 ± 0.2	0.063
PVDF + 2 wt% (ref. 2)	139.3	24.0 ± 0.3	0.080
PP + 2 wt% (ref. 2)	12.1	47.2 ± 1.9	0.027
PC + 2 wt% (ref. 2)	1.0	34.8 ± 0.3	0.001
ABS + 2 wt% (ref. 2)	14.8	−34.2 ± 0.7	0.017
PA6 + 2 wt% (ref. 2)	3.8	−56.3 ± 2.9	0.012
PARA + 2 wt% (ref. 2)	2.4	−46.6 ± 1.3	0.005
PA66 + 2 wt% (ref. 2)	28.2	−29.5 ± 1.4	0.025
Epoxy + 1 wt% (ref. 40)	2	−25.5 ± 0.2	0.002
PEEK + 1 wt% (ref. 8)	6.2	48.0 ± 1.3	0.014
PEEK + 3 wt% (ref. 41)	21.8	36.2 ± 0.1	0.028
PMMA + 2 wt% (ref. 7)	57.2	53.4 ± 2.2	0.163
PCL + 2 wt% (ref. 9)	98.3	38.1 ± 1.6	0.143
PLA + 2 wt% (ref. 9)	177.3	43.5 ± 2.3	0.336
TPU 1185A10 + 2 wt% (this study)	13.7	38.1 ± 4.0	0.020

Finally, the relative composition of the TPU matrices was expressed by the area fraction *R* of each functional group, namely urethane, ester, and ether, with respect to the sum of all evaluated functional-group band areas. For this purpose, the integrated areas obtained from the carbonyl stretching region and the C–O–C stretching region of ether groups were combined. The resulting relative functional-group compositions are shown in Fig. 8.

The ternary plot in Fig. 9 reveals a dependence of the Seebeck coefficient on the chemical composition of the TPU matrices. The urethane-rich matrix C74D50 yields the lowest Seebeck coefficient, approximately 22 µV K^−1^, whereas matrices with higher ester or ether contributions show a substantially no pronounced decrease or even increase in the case of relative high ether content. These results indicate that urethane functionalities have a significant decreasing impact on the thermoelectric response of SWCNT-filled TPU composites than ester- or ether-rich matrix segments. Based on this internally referenced spectral evaluation, systematic differences in the relative urethane, ester, and ether contributions were observed and indicate a composition-dependent trend.

**Fig. 9:**
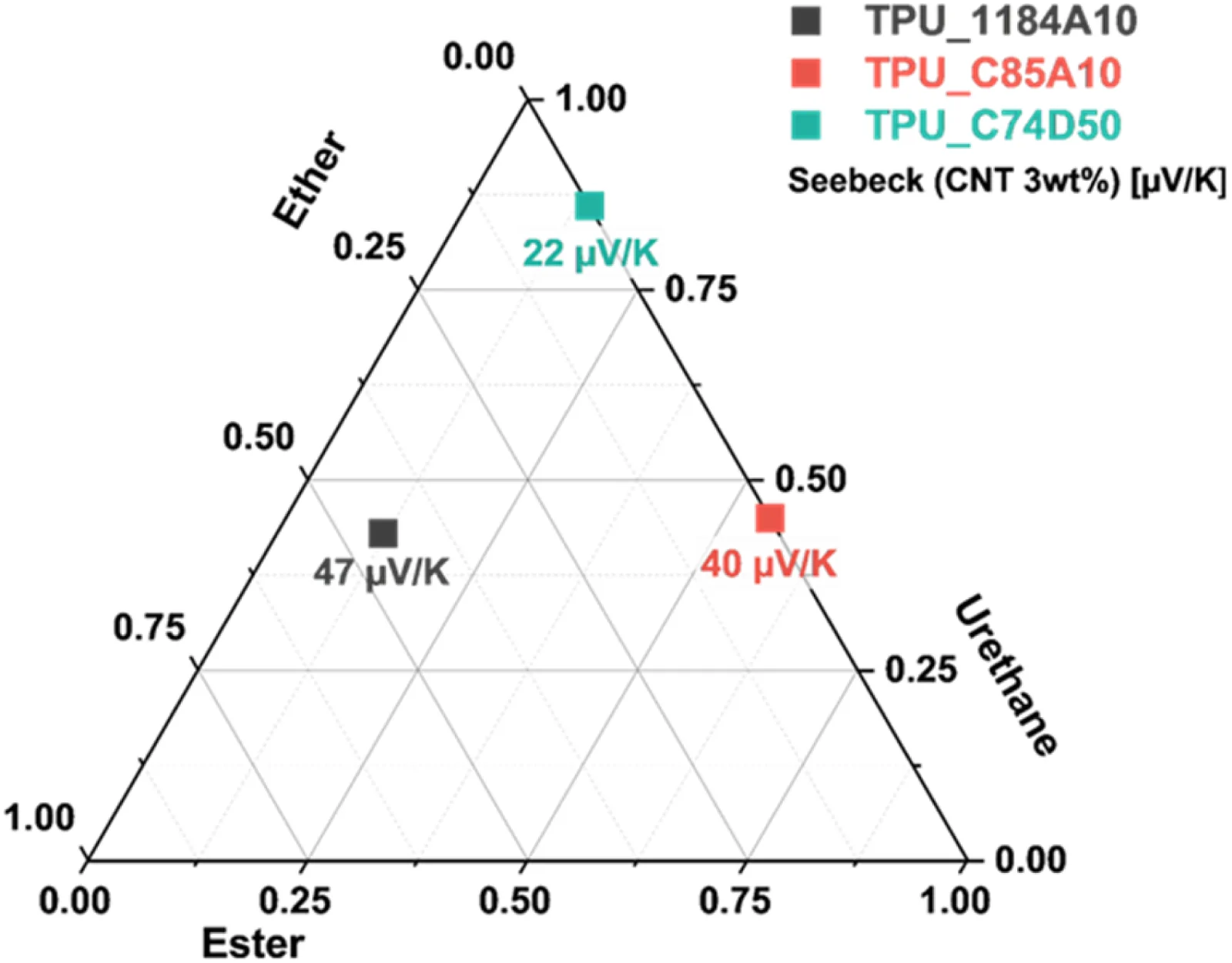
Ternary plot of the relative urethane, ester, and ether contributions of the TPU matrices, together with the Seebeck coefficients measured for composites containing 3 wt% SWCNT.

This behavior may originate from specific interfacial interactions between the TPU matrices and the Tuballs. A plausible explanation is that urethane-associated polar groups alter the charge-carrier balance in the Tuballs's network, for example through interfacial dipoles or weak interfacial donor–acceptor and inductive interactions, charge-carrier density, or energy filtering at the filler–matrix interface. These interactions reduce the p-type character of Tuballs and consequently decrease the positive Seebeck coefficient. Accordingly, the matrix chemistry may provide an additional parameter for tuning the thermoelectric properties of SWCNT/polymer composites.

Nevertheless, this mechanistic assignment remains hypothetical at this stage. Further model-based analysis and complementary experimental evidence are required to verify whether the observed reduction in the Seebeck coefficient is primarily caused by altered electronic interactions at the SWCNT-matrix interface, may be attributed to orientation phenomena of hard and soft segments of matrix chains or by changes in SWCNT dispersion and network morphology. The following study will give deeper insights into ester and ether-free polymeric urethane model systems.

## Conclusions

In this study, our aim was to evidence the hypothesis that nitrogen-containing groups in polyurethanes influence the thermoelectric properties of electrically conductive composites. We show that the urethane content of TPU systematically alters the Seebeck coefficient of SWCNT networks, indicating an interfacial doping-like effect. To this purpose, three different types of commercial TPUs were investigated using a practical model system. Two TPUs with low Shore hardness were selected, which, however, differ in their proportion of soft segments consisting of polyester (C85A10) or polyether groups (1185A10). The third TPU (C74D50) exhibited a higher Shore hardness, which roughly correlates with a higher proportion of urethane-based hard segments and contains mainly polyester-based soft segments. As the manufacturer provides no information on the exact composition of the TPUs, IR measurements, which are relatively straightforward to perform, were used to identify the structure and fundamentally understand the observed thermoelectrical property changes.

Transmission microscopy revealed that the macroscopic distribution of SWCNTs in the polymer matrices is quite similar. Some of the incorporated SWCNTs formed relatively large, elongated agglomerates. Transmission microscopy revealed that the macroscopic distribution of SWCNTs in the matrices is comparable and could not be the reason for the impact on the thermoelectric properties.

Quantitative IR measurements revealed that the urethane content in TPU-C74D50 was more than twice as high as in the other two TPU types. Only ester groups were detected as the soft segment in TPUs C74D50 and C85A10, with the ester content in C85A10 being 3–4 times higher than in C74D50. In TPU-1185A10, a similarly low ester as in C74D50 was calculated. Ether groups were detected only in TPU-1185A10, where their proportion was four times higher than the ester content.

Additional analytical approaches, such as elemental analysis (CHN), titrations after digestion or photometric determination can be used to quantitatively estimate the actual nitrogen content of TPUs. However, the differences in nitrogen-related functionality were considered here as relative trends derived from their chemical formulation and supported by FTIR analysis. In this study, emphasis is placed on the interfacial role of urethane groups in the SWCNT-filled TPUs rather than on an absolute bulk compositional nitrogen determination.

A comparison of the measured Seebeck coefficients shows a strong deviation only between the soft TPU grades (1185A10, C85A10) and the harder C74D50 grade. The Seebeck coefficient for the two TPUs (C85A10, 1185A10) with a higher proportion of soft segments was determined to be around 40–47 µV K^−1^. The different chemical structures of the ester and ether groups result in a slight change in the *S*-value. So, the Seebeck coefficient of 1185A10, having the highest ether content, was measured at 47 µV K^−1^ whereas C85A10 achieved a lower *S*-value of 40 µV K^−1^. It can be assumed that the ether groups in TPU lead to a slightly p-doping of Tuball's compared to the *S*-value of Tuball powder. However, for C74D50, which has a high urethane content, Seebeck coefficients of only 10 µV K^−1^ were measured at higher SWCNT contents, which corresponds to an n-doping effect on the thermoelectric properties. It can be concluded that the urethane group exerts a more dominant influence on the thermoelectric properties than the ester and ether groups. This result supports the hypothesis that nitrogen-containing groups can cause a shift in the Seebeck coefficient towards lower, and possibly negative, *S*-values.

For further studies on this effect, neat polyurethanes and mixtures with additional reactive diamine-functional groups are to be investigated in order to systematically vary the number of nitrogen-containing functional groups with electron–donor properties. The research should remain as closely aligned as possible with practical, implementable solutions.

At all, the study introduces a non-covalent interface-engineering strategy to tune the thermoelectric response of SWCNT/polymer composites through the intrinsic chemistry of thermoplastic polyurethane. Using the urethane content as an internal molecular parameter, the Seebeck coefficient of SWCNT networks can be systematically modulated without additional chemical functionalization or doping of the nanotubes. This approach preserves the electronic integrity of the carbon phase, avoids additional synthesis steps, and is compatible with and scalable for melt processing. It therefore offers a simple, recyclable, and industrially relevant route toward flexible carbon–polymer thermoelectric materials.

## Author contributions

Conceptualization, B. K.; methodology, B. K., C. Z.; software, B. K.; validation, B. K., C. Z.; formal analysis, B. K., C. Z.; investigation, B. K., C. Z.; resources, B. K., C. Z.; data curation, B. K., C. Z.; writing – original draft preparation, B. K., C. Z.; writing – review and editing, B. K., C. Z.; visualization, B. K., C. Z.; supervision, B. K., C. Z.; all authors have read and agreed to the published version of the manuscript.

## Conflicts of interest

There are no conflicts to declare.

## Supplementary Material

RA-016-D6RA06192C-s001

## Data Availability

The raw data for all measurements performed for this article are available at zenodo at https://doi.org/10.5281/zenodo.20932248. Supplementary information (SI): Table S1: general integration ranges for sub band evaluation of ester, urethane and ether groups; Table S2: IR-fit methods and results. See DOI: https://doi.org/10.1039/d6ra06192c.
