# Supplementary material for: Nitrogen content governs thermoelectric performance in TPU/SWCNT composites
Source: RSC Adv. 2026 Oct 5;16(46):60228–38. doi: 10.1039/d6ra06192c (PMC13637523; doi:10.1039/d6ra06192c)
Supplement: Supplementary file 1 [file RA-016-D6RA06192C-s001.pdf]

Supplementary Information (SI) for RSC Advances.
This journal is © The Royal Society of Chemistry 2026
Supplementary Information (SI) for RSC Advances.
This journal is © The Royal Society of Chemistry

Supporting Information

Nitrogen Content Governs Thermoelectric Performance in TPU/SWCNT Composites

Cordelia Zimmerer[1], Beate Krause[2]*

[1] Leibniz-Institut für Polymerforschung Dresden e.V. (IPF), Division Polymer Materials Engineering, Hohe Str. 6, 01069 Dresden, Germany; zimmerer@ipfdd.de (C.Z.)
[2] Leibniz-Institut für Polymerforschung Dresden e.V. (IPF), Division Macromolecular Chemistry, Hohe Str. 6, 01069 Dresden, Germany, krause-beate@ipfdd.de (B.K.)
* Correspondence: Beate Krause, krause-beate@ipfdd.de; Tel.: +49-351-4658-736

The raw data for this manuscript were published as an open-access dataset:

Krause, Beate, and Cordelia Zimmerer.
Raw Data for the Paper "Nitrogen Content Governs Thermoelectric Performance in TPU/SWCNT Composites".
Zenodo, June 26, 2026. https://doi.org/10.5281/zenodo.20932248

This study reports IR spectroscopy measurements of three different polyurethanes to characterize the relative chemical composition and proportion of urethane and ester or ether groups present in each of the polymeric backbones.

Table S1: General integration ranges for sub band evaluation of ester, urethane and ether groups

**Integrated spectral range for functional groups**	**Urethane, vNH**	**Urethane vC=O**	**Ester, v C=O**	**Ether, vC-O**
**Wave number range [cm^{-1}]**	3495-3210	1728.5-1640	1780-1695	1160-1025

Table. S2: FTIR-Fit methods and results

Band shape	Position [cm^{-1}]	Intensity [a.u.]	Width [a.u.]	Integral [a.u.]	
**1185A10 TPU_C85A10_3-22_ATR_DLaTGS_26.01.2023_2BSL.0**					
Number of bands: 3<br>Algorithm: Local Least Squares<br>x-Start: 1825.0; x-End: 1610.0<br>RMS- error (locales residuum): 0.055664					
93%Lorentz+Gauss	1700.55	1.98	21.67	65.76	urethane
35%Lorentz+Gauss	1713.68	0.35	16.64	7.37	urethane
21%Lorentz+Gauss	1730.49	0.97	15.56	17.67	ester
Number of bands: 6<br>Algorithm: Local Least Squares<br>x-Start: 1174.2; x-End: 999.6<br>RMS- error (locales residuum): 0.011066					
0%Lorentz+Gauss	1017.45	0.49	8.78	4.54	
47%Lorentz+Gauss	1036.24	1.57	28.67	21.25	urethane
11%Lorentz+Gauss	1063.99	1.40	27.88	43.64	urethane
10%Lorentz+Gauss	1078.60	0.44	13.77	6.79	urethane
8%Lorentz+Gauss	1104.07	1.64	38.32	69.75	ether
**74D50 TPU_C74D50_3-22_ATR_DLaTGS_26.01.2023_1BSL.1**					
Number of bands: 4<br>Algorithm: Levenberg-Marquardt<br>x-Start: 1825.0; x-End: 1610.0<br>RMS- error (locales residuum): 0.285267					
96%Lorentz+Gauss	1697.00	6.51	34.10	344.52	urethane
0%Lorentz+Gauss	1714.99	1.66	21.69	38.35	urethane
0%Lorentz+Gauss	1731.00	3.02	18.41	59.09	ester
0%Lorentz+Gauss	1740.00	0.30	8.30	2.61	ester
Number of bands: 6<br>Algorithm: Levenberg-Marquardt<br>x-Start: 1105.3; x-End: 999.6<br>RMS- error (locales residuum): 0.019021					
0%Lorentz+Gauss	1017.86	0.26	7.77	2.14	
100%Lorentz+Gauss	1036.28	0.20	19.04	5.88	urethane
100%Lorentz+Gauss	1055.96	0.66	29.53	30.74	urethane
12%Lorentz+Gauss	1067.60	0.43	17.61	8.50	urethane
0%Lorentz+Gauss	1078.11	0.39	11.65	4.86	urethane
0%Lorentz+Gauss	1085.01	0.21	7.53	1.71	
**C85A10 TPU_C85A10_3-22_ATR_DLaTGS_26.01.2023_2BSL.0**					
Number of bands: 4<br>Algorithm: Local Least Squares<br>x-Start: 1825.0; x-End: 1610.0<br>RMS- error (locales residuum): 0.055664					
88%Lorentz+Gauss	1700.29	4.43	26.84	179.19	
47%Lorentz+Gauss	1713.19	1.52	18.95	37.64	urethane
7%Lorentz+Gauss	1727.14	4.36	17.37	83.44	ester
14%Lorentz+Gauss	1738.49	1.59	16.13	29.20	ester
Number of bands: 6<br>Algorithm: Local Least Squares					

x-Start: 1105.3; x-End: 999.6<br>RMS- error (locales residuum): 0.027081					
100%Lorentz+Gauss	1016.69	0.26	30.41	12.77	
42%Lorentz+Gauss	1036.51	0.10	12.88	1.65	urethane
50%Lorentz+Gauss	1048.05	0.21	31.56	8.86	urethane
87%Lorentz+Gauss	1061.57	0.54	26.51	21.76	urethane
62%Lorentz+Gauss	1076.71	0.60	20.98	17.44	urethane
35%Lorentz+Gauss	1082.86	0.14	9.69	1.66	
